# Pure dysarthria and dysarthria-facial paresis syndrome due to internal capsule and/or corona radiata infarction

**DOI:** 10.1186/s12883-015-0439-5

**Published:** 2015-10-07

**Authors:** Koji Tanaka, Takeshi Yamada, Takako Torii, Takeo Yoshimura, Kei-ichiro Takase, Osamu Togao, Yoshifumi Wakata, Akio Hiwatashi, Naoki Nakashima, Jun-ichi Kira, Hiroyuki Murai

**Affiliations:** Department of Neurology, Neurological Institute, Graduate School of Medical Sciences, Kyushu University, 3-1-1 Maidashi, Higashi-ku, Fukuoka, 812-8582 Japan; Department of Neurology, Saiseikai Fukuoka General Hospital, 1-3-46 Tenjin, Chuo-ku, Fukuoka, 810-0001 Japan; Department of Neurology, Fukuoka City Hospital, 13-1 Yoshizukahonmachi, Hakata-ku, Fukuoka, 812-0046 Japan; Department of Neurology, Iizuka Hospital, 3-83 Yoshiomachi, Iizuka, Fukuoka, 820-8505 Japan; Department of Clinical Radiology, Graduate School of Medical Sciences, Kyushu University, 3-1-1 Maidashi, Higashi-ku, Fukuoka, 812-8582 Japan; Medical Information Center, Kyushu University Hospital, 3-1-1 Maidashi, Higashi-ku, Fukuoka, 812-8582 Japan; Department of Neurological Therapeutics, Graduate School of Medical Sciences, Kyushu University, 3-1-1 Maidashi, Higashi-ku, Fukuoka, 812-8582 Japan

**Keywords:** Cerebral small vessel diseases, Ischemic stroke, Diffusion magnetic resonance imaging, Dysarthria, Facial paresis

## Abstract

**Background:**

Pure dysarthria (PD) and dysarthria-facial paresis syndrome (DFP) mainly result from lenticulostriate artery territory infarction. PD and DFP are rare clinical entities, often grouped without distinction. The purpose of this study was to examine clinical and radiographic differences between PD and DFP due to unilateral internal capsule and/or corona radiata infarction.

**Methods:**

Using a database that included consecutive patients with ischemic stroke admitted to the neurological stroke units of three hospitals within 7 days from onset between September 2011 and April 2014, we retrospectively extracted first-ever stroke patient data, who presented with PD or DFP with a single ischemic lesion localized in the internal capsule and/or corona radiata. Patients with weakness, ataxia, sensory deficit, or cortical symptoms were excluded. Ischemic lesion volume was calculated by the ABC/2 method on diffusion-weighted imaging (DWI). DWI images were normalized and superimposed to the template for PD and DFP. We compared patients' characteristics between PD and DFP.

**Results:**

A total of 2126 patients, including 65 patients (3.1 %) with PD or DFP, were registered. Of these, 13 PD patients and 18 patients with DFP due to unilateral internal capsule and/or corona radiata infarction were included for analysis. Compared with DFP patients, PD patients had longer onset-to-door time (median 37.5 *vs.* 10.8 h, *p* = 0.031), shorter vertical length (C component) of ischemic lesions (median 12.0 *vs.* 18.8 mm, *p* = 0.007), and smaller ischemic lesion volume (median 285 *vs.* 828 mm^3^, *p* = 0.023). Ischemic lesions causing PD were located more frequently in the left hemisphere than DFP (92 % *vs.* 56 %, *p* = 0.045). The superimposed lesion pattern indicated that DFP had lesions more medial and involving posterior portions of the putamen and the caudate body, as well as more of the genu and posterior limb of the internal capsule, than PD. Ninety days after onset, symptoms disappeared in 21 (72 %) out of 29 patients.

**Conclusions:**

In cerebral infarction limited to the internal capsule and/or corona radiata, PD is derived from smaller and left-sided lesions with delay in diagnosis compared with DFP. The clinical course of those with PD and DFP might be benign.

## Background

Pure dysarthria (PD), the sudden onset of unaccompanied dysarthria from a vascular insult, was originally described as a lacunar syndrome [1]. Ozaki et al. [2] also reported that PD was caused by lesions in the internal capsule and/or corona radiata. PD was subsequently described as an extreme of dysarthria-facial paresis syndrome (DFP), a variant of dysarthria-clumsy hand syndrome [3]. Although PD and DFP caused by unilateral brain infarction are well-established entities, they are often grouped without distinction. The aim of this study was to examine the clinical and radiographic differences between patients with PD and DFP, especially due to internal capsule and/or corona radiata infarction, the most common causes of PD and DFP [2–5].

## Methods

This study used data retrospectively extracted from a prospective, observational, multicenter registry, which is part of the broad-area, network-based project to drive clinical research at Kyushu University Hospital. The methods of our registry have been previously described in detail [6]. In brief, the database enrolled all consecutive patients with acute ischemic stroke admitted to the neurological stroke units of three hospitals (Saiseikai Fukuoka General Hospital, Fukuoka City Hospital, Iizuka Hospital) within 7 days from onset. Each local ethics committee (Kyushu University Hospital, Saiseikai Fukuoka General Hospital, Fukuoka City Hospital, and Iizuka Hospital) approved the study, and patient clinical data were submitted from the study office in each facility to the data center in Kyushu University Hospital. Between September 2011 and April 2014, patients who fulfilled the following criteria were extracted from the database: (1) presenting PD or DFP upon admission; (2) no previous history of stroke; and (3) an acute single ischemic lesion in the internal capsule and/or corona radiata in the territory of the lenticulostriate artery confirmed on diffusion-weighted imaging (DWI) upon admission.

All patients received neurological assessment upon admission. Dysarthria was reported when clinically apparent upon neurologic examination, which corresponds to the National Institutes of Health Stroke Scale subscore (item 10) of 1 or higher. PD was defined as dysarthria of sudden onset without any other significant symptoms or signs of neuropsychological abnormalities, including aphasia, motor weakness, ataxia, sensory loss, or cranial nerve dysfunction, except for those related to articulation [4], and not accompanied by facial paresis. DFP was defined as PD combined with supranuclear facial paresis. The patient’s clinical background characteristics, including sex, age, cardiovascular risk factors including hypertension, diabetes mellitus, dyslipidemia, and atrial fibrillation, onset-to-door time, and onset-to-imaging time were collected from medical charts. Patients were routinely reassessed at 90 days from onset by an attending neurologist in the outpatient clinic. If the patient could not visit the clinic, follow-up was performed by telephone interview or by a mail-in survey.

All MRI studies, including DWI and time-of-flight MRA, were performed on one of the three 1.5-T echo-planar imaging equipped clinical whole body scanners (Gyroscan NT intera [*n* = 3], Philips Medical Systems, Best, the Netherlands; Magnetom Avanto [*n* = 12], Siemens Medical Solutions, Erlangen, Germany; GE Signa [*n* = 16], GE Medical Systems, Milwaukee, WI, USA). MRI protocols were not entirely uniform in each center, but all included axial DWI using single-shot echo-planar imaging (repetition time 2875–6000 ms; echo time, 70–100 ms; flip angle, 90°; matrix, 256 × 256; b values of 1000 s/mm^2^; slice thickness, 5 mm; inter-slice gap, 1–1.5 mm). MRA and carotid ultrasound were performed on all patients to assess atherosclerotic lesions of cervicocephalic arteries. DWI data were transferred to the coordinating center and analyzed using image analysis software (ImageJ version 1.48; National Institute of Health, Bethesda, MD, USA) by investigators blinded to clinical information. The infarct volume on DWI was calculated by the ABC/2 method [7, 8]. The slice with the largest lesion was visually selected and the longest lesion axis, x (A), on this slice was measured. A second line, y (B), was drawn perpendicular to the first line at the widest dimension. The z (C) axis was computed by multiplying the number of slices by slice thickness including inter-slice gap. Afterwards, images were normalized to the Montreal Neurological Institute (MNI) space using SPM8 software (Wellcome Department of Cognitive Neurology, London, UK) based on MATLAB version R2013b (The Mathworks, Sherborn, MA, USA) [9, 10]. Lesion plots were created on a voxel-by-voxel basis indicating the infarction frequency in each voxel. Color-coded lesion plots at the level of internal capsule and corona radiata were superimposed on the normalized T1-weighted image template.

Statistical analysis was performed using statistical software (JMP version 9.0; SAS Institute Inc., Cary, NC, USA). Results are expressed as mean ± standard deviation, or median (inter-quartile range). Differences in continuous variables were assessed using the Student t-test or Mann–Whitney U test, as applicable. Differences in categorical variables were assessed using Fisher’s exact tests. Clinical characteristics and radiographic parameters were compared between patients with PD and DFP. All probability values reported are two-sided, and probability values < 0.05 were considered significant.

## Results

A total of 2126 patients (1242 males, 73.3 ± 12.6 years) with acute ischemic stroke were registered during the study period. Of these, 65 patients (3.1 %) with PD or DFP were eligible, while 34 were excluded because of history of stroke (*n* = 8), multiple ischemic lesions (*n* = 13), ischemic lesions other than the internal capsule and corona radiata (*n* = 12), or not detected (*n* = 1) on DWI. Lesion topography of all 65 patients is shown in Table 1. Finally, 13 patients with PD and 18 patients with DFP, who met the criteria, were included for analysis. All patients were right-handed, and the degree of dysarthria was mild to moderate. None had atrial fibrillation or carotid artery stenosis of > 50 % or occlusion.

**Table 1:** Lesion topography of all 65 patients presenting with pure dysarthria and dysarthria-facial paresis syndrome

	PD (*n* = 27)		DFP (*n* = 38)	
Topography of lesions^a^	Right	Left	Right	Left
Cortex/subcortex	1	2	4	5
Deep white matter	4	5	3	2
Internal capsule/corona radiata	1	13	11	13
Putamen/pallidum				2
Insular lobe	1	1	1	
Pons	3	1		1

PD, pure dysarthria; DFP, dysarthria-facial paresis syndrome

^a^Eight patients had more than one anatomical territory involved on diffusion-weighted imaging

**Fig. 1:**
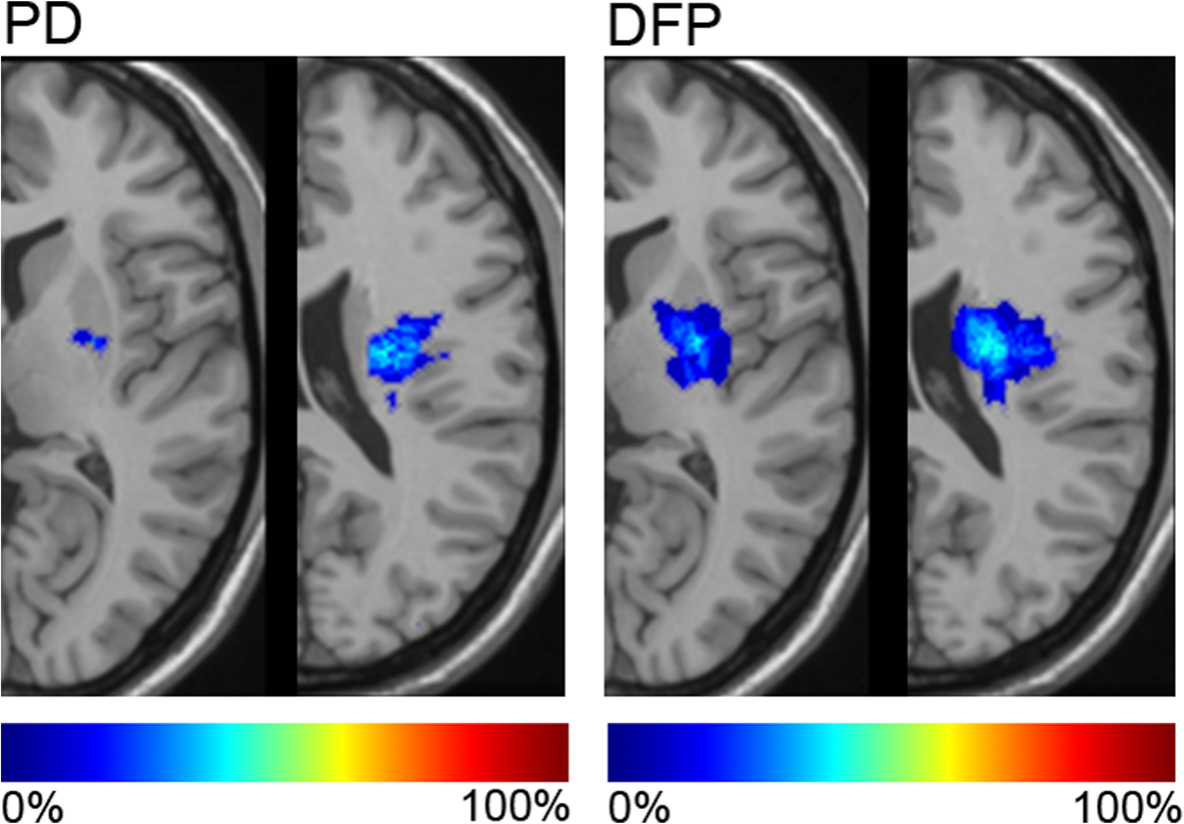
Color-coded lesion overlay plots. Lesion plots calculated from DWI images were superimposed on T1-weighted templates. Each axial slice at the level of internal capsule (left) and corona radiata (right) was given for PD (pure dysarthria) and DFP (dysarthria-facial paresis syndrome). The lesion side was not distinguished

Patients' characteristics are shown in Table 2. Compared with DFP patients, PD patients had a longer onset-to-door time (median 37.5 [16.5–51.0] h *vs.* 10.8 [7.3–27.1] h, *p* = 0.031). Although two patients with DFP arrived at hospitals within the time for thrombolysis indication, they did not undergo thrombolysis due to too mild neurological deficits. Median onset-to-imaging time was 38.5 hours in PD and 11.8 hours in DFP (*p* = 0.029). From image analyses, PD had a shorter vertical length (C component) of ischemic lesions (median 12.0 [6.0–13.0] mm *vs.* 18.8 [12.0–20.6] mm, *p* = 0.007), and a smaller ischemic lesion volume (median 285 [136–403] mm^3^*vs.* 828 [291–1664] mm^3^, *p* = 0.023) on DWI. Ischemic lesions causing PD were located more frequently in the left hemisphere than those causing DFP (92 % *vs.* 56 %, *p* = 0.045). The superimposed DWI lesion pattern indicated that at the level of corona radiata, lesions in DFP were more medial and involved the posterior portions of the caudate body than those in PD. Both PD and DFP were derived from rather the anterior part of the corona radiata, not adjacent to the lateral ventricle. DFP lesions also involved more of the genu and posterior limb of the internal capsule, and the posterior portion of the putamen than PD lesions at the level of internal capsule (Fig. 1).

**Table 2:** Characteristics of patients with PD and DFP due to internal capsule and/or corona radiata infarction

	Total (*n* = 31)	PD (*n* = 13)	DFP (*n* = 18)	*p*-value
Male, *n* (%)	19 (61)	9 (69)	10 (56)	0.484
Age, years (mean ± SD)	68.1 ± 11.6	66.5 ± 11.1	69.2 ± 12.1	0.534
Hypertension, *n* (%)	26 (84)	10 (77)	16 (89)	0.625
Diabetes mellitus, *n* (%)	10 (32)	5 (38)	5 (28)	0.701
Dyslipidemia, *n* (%)	9 (29)	2 (15)	7 (39)	0.237
Onset-to-door time, hours, median (IQR)	21.0 (8.0–45.0)	37.5 (16.5–51.0)	10.8 (7.3–27.1)	0.031
Onset-to-imaging time, hours, median (IQR)	22.5 (10.0–46.5)	38.5 (17.8–53.5)	11.8 (9.0–28.1)	0.029
DWI parameters, median (IQR)				
A component, mm	12.1 (7.7–14.4)	9.0 (6.5–14.1)	12.7 (9.3–14.6)	0.230
B component, mm	5.8 (4.7–9.5)	5.0 (4.3–9.0)	6.2 (5.2–10.6)	0.128
C component, mm	13.0 (6.5–19.5)	12.0 (6.0–13.0)	18.8 (12.0–20.6)	0.007
Ischemic lesion volume, mm^3^	379 (175–1167)	285 (136–403)	828 (291–1664)	0.023
Side of lesion, left, *n* (%)	22 (71)	12 (92)	10 (56)	0.045

SD, standard deviation; IQR, interquartile range; PD, pure dysarthria; DFP, dysarthria-facial paresis syndrome; DWI, diffusion-weighted imaging

Twenty-nine patients (94 %) had catamnestic follow-up at 90 days. Symptom progression after admission was seen in one PD case, which worsened to develop DFP. One DFP case due to right corona radiata infarction developed pure motor hemiparesis as a result of recurrence in the same region, while symptoms remained in seven patients, and disappeared in 21 (72 %).

## Discussion

This study demonstrated that approximately 3 % of patients with acute ischemic stroke present with PD or DFP upon admission. Half of those symptoms were derived from the ischemic lesion limited to the internal capsule and/or corona radiata. Both PD and DFP, as atypical lacunar syndromes, could also be derived by events other than lacunar infarct [11], with previous reports indicating several culprit ischemic lesions causing PD or DFP in the cortices, brainstem, or cerebellum [3, 12–17], rather than the internal capsule and/or corona radiata. Although we found some ischemic lesions in regions other than the internal capsule and/or corona radiata, the locations of many of the lesions indicated an embolic mechanism. Therefore, it is unclear whether lesions outside of the internal capsule and/or corona radiata were responsible for PD or DFP.

In cerebral infarction limited to the internal capsule and/or corona radiata, PD patients exhibited smaller and left-side preponderant lesions compared with DFP. The infarct size influences the clinical presentation of PD and DFP, as for classical lacunar syndromes; patients with motor weakness had a larger infarct size, which involved the corticospinal tract, compared with those without [6]. Furthermore, because the cortico-bulbar tract for articulation is functionally left-side predominant [18], dysarthria may result from small lacunar lesions on the left side, but rarely from those on the right. Laterality of cortico-bulbar tract lesions in PD may be masked by facial paresis in DFP, because facial paresis equally impairs labial sounds, independent of lesion sides.

In the present study, PD patients had longer onset-to-door time than DFP patients. Facial drop and speech difficulty are included in “FAST,” derived from the Cincinnati Prehospital Stroke Scale [19, 20], both of which are associated with urgent medical consultation [21]. Our data suggest a synergistic effect of individual symptoms on patient’s medical-seeking behavior.

The clinical course of PD and DFP in our study was benign, as previously reported for ‘atypical’ lacunar syndrome, including DFP and PD as the most frequently presenting forms [22]. Progressive motor deficits lead to poor functional outcome in patients with acute lacunar infarction located in the lenticulostriate artery territory. Ohara et al. [23] reported that lacunar infarct located in the posterior region of the corona radiata adjacent to the lateral ventricle predicts progressive motor deficits. Our lesion overlay plots suggest that PD and DFP were less frequently caused by ‘posterior type infarct.’ Although our results also indicate that DFP involved a part of the basal ganglia, it was not clear if the basal ganglia involvement impacted the clinical manifestation or clinical course in DFP.

This study has several limitations. First, the retrospective design might contribute to recorder/investigator bias. Second, the small number of cases may introduce statistical error. Third, DWI parameters may be affected by specific MRI protocols, which vary between individual centers, or differences in onset-to-door time between PD and DFP. Moreover, anatomical structures in the brain are not completely consolidated by normalization into the MNI space. Finally, the association between the laterality of ischemic lesions and the dominant hemisphere of speech processing was not clear, as the handedness was only confirmed by individual interviews, not by specific inventories.

## Conclusions

In cerebral infarction of the internal capsule and/or corona radiata, PD is derived from smaller and left-sided ischemic lesions with delay in diagnosis compared with DFP. The clinical course of PD and DFP patients might be benign.
